# Self-steering partially coherent beams

**DOI:** 10.1038/srep39957

**Published:** 2017-01-04

**Authors:** Yahong Chen, Sergey A. Ponomarenko, Yangjian Cai

**Affiliations:** 1 College of physics, Optoelectronics and Energy & Collaborative Innovation Center of Suzhou Nano Science and Technology, Soochow University, Suzhou 215006, China; 2 Key Lab of Advanced Optical Manufacturing Technologies of Jiangsu Province & Key Lab of Modern Optical Technologies of Education Ministry of China, Soochow University, Suzhou 215006, China; 3 Department of Electrical and Computer Engineering, Dalhousie University, Halifax, Nova Scotia B3J 2X4, Canada

## Abstract

We introduce a class of shape-invariant partially coherent beams with a moving guiding center which we term self-steering partially coherent beams. The guiding center of each such beam evolves along a straight line trajectory which can be engineered to make any angle with the *x*-axis. We show that the straight line trajectory of the guiding center is the only option in free space due to the linear momentum conservation. We experimentally generate a particular subclass of new beams, self-steering Gaussian Schell beams and argue that they can find applications for mobile target tracing and trapped micro- and/or nanoparticle transport.

Optical trapping is a powerful tool for manipulation of micro-particles or micro-targets. Since the seminal work of Ashkin^1^ on trapping a particle through using radiation forces exerted by a Gaussian laser beam, the technique of optical trapping has been developed and is now widely applied in a variety of fields to manipulate micro-sized dielectric particles, cells, DNA and RNA molecules, neutral atoms, and living biological cells^2^,^3^,^4^,^5^,^6^. At the same time, it is well established now that under certain conditions, partially coherent sources can generate highly directional light beams with the same far-field intensity distributions as do fully coherent laser beams^7^,^8^. In this connection, it was found^9^ that partially coherent sources can give rise the same optical forces as those due to laser beams at any output plane of a generic ABCD optical system. Further, the same authors showed that partially coherent beams are superior to the laser beams in trapping biological samples because the former generate less thermal heating than the latter. To date, certain types of partially coherent beams have been shown to be especially beneficial in trapping neutral micro-particles^10^,^11^,^12^,^13^,^14^.

In this paper, we demonstrate that the evolution of a beam guiding center position **R**_c_ (*z*), defined as


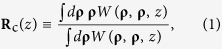


plays an important role in tracking and manipulating micro- and/or nanoparticles with partially coherent beams. In Eq. (1), *W*(**ρ**_1_, **ρ**_2_, *z*) is a cross-spectral density (CSD) of the beam field at a pair of points **ρ**_1_ and **ρ**_2_ in the transverse plane *z.*^15^. We notice that all partially coherent beams known to date have stationary guiding centers, which effectively precludes their use for transporting trapped particles. It is therefore instructive to explore the possibility of engineering shape-invariant partially coherent beams with mobile guiding centers which would enable one to transport trapped particles along prescribed trajectories. Such steering partially coherent beams can also find applications for tracing mobile military, meteorological and other targets.

We show how any shape-invariant partially coherent beam can be engineered into a self-steering one with its guiding center traveling along a straight line trajectory. The self-steering beam trajectory makes an angle with the *x*-axis that can be controlled by adjusting the beam phase at the source. We experimentally implement a subclass of new beams, self-steering Gaussian Schell (SSGS) beams and show that the lower the SSGS beam coherence the less susceptible the beam to speckle and/or spurious fringe formation on its propagation.

## Results

### Theory of self-steering partially coherent beams

We start by noticing that the intensity of any shape-invariant partially coherent beam is self-similar and can be related to the source intensity as^16^


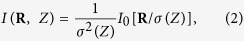


where *I*_0_[**R**] is the source intensity distribution, **R** ≡ (*X, Y*) is a dimensionless radius vector with *X* = *x*/*σ*_*I*_ and *Y* = *y*/*σ*_*I*_, **ρ** ≡ (*x, y*) is a radius vector in the plane transverse to the beam propagation direction, *σ*_*I*_ denotes the beam waist, and *σ*(*Z*) is a propagation factor depending on beam intensity and coherence distributions at the source. We note that the intensity amplitude scaling in Eq. (2) guarantees beam power conservation,


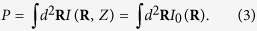


According to the complex Gaussian representation^17^, the CSD of any partially coherent beam at the source can be expanded into a complete set of pseudo-modes as^18^,


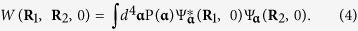


Here P (**α**) is a nonnegative distribution function that guarantees non-negative definiteness of *W* and {Ψ_**α**_(**R**, 0)} are the complex Gaussian pseudo-modes. Further, 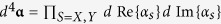 and **α** = (**u** + *i***v**)/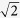. The pseudo-modes {Ψ_***α***_(**R**, 0)} are given by the expression


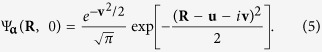


Consider now a shifted P− distribution such that


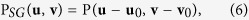


where P is the distribution of an original shape-invariant source. We will now demonstrate that this engineered partially coherent source gives rise to a self-steering shape-invariant beam.

Upon free-space propagation each pseudo-mode Ψ_**α**_(**R**, 0) satisfies the paraxial wave equation which can be readily solved yielding^17^,^19^,


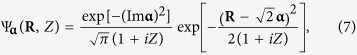


where *Z* = *z*/*z*_*R*_ with 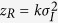, *z* being the propagation distance and *k* being the wavenumber. We stress here that *Z* is a normalized variable. The CSD in the *Z* plane can then be expressed as


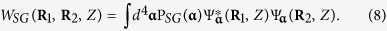


It follows at once from Eqs (6) and (8) that the self-steering beam intensity, *I*_*SG*_(***R**, z*) ≡ *W*_*SG*_(***R**, **R**, Z*), is given by


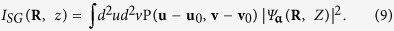


Here


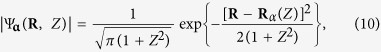


and the guiding center of each mode evolves according to **R**_α_(*Z*) = **u** + *Z***v**. Thus, the self-steering beam intensity can be viewed as an incoherent superposition of weighted Gaussian intensities with various guiding center positions. On substituting from Eq. (10) into (9), shifting the integration variables, and comparing with Eq. (4), evaluated at the same spatial point, one can see that the intensity profile of the beam is maintained up to a scaling factor such that


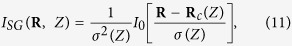


where **R**_c_ (*Z*) = **u**_0_ + *Z***v**_0_ which proves our assertion that any shape-invariant source with a shifted P− distribution generates the corresponding self-steering beam. Our result is completely general, except we require the knowledge of both the intensity and coherence distribution at the source to determine the explicit form of the propagation factor *σ*(*Z*).

To illustrate our general results, we consider the familiar case of a Gaussian Schell model (GSM) source whose shifted P− distribution can be inferred from ref. 17 as


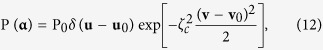


where P_0_ is a nonnegative constant, and *ζ*_*c*_ = *σ*_*c*_/*σ*_*I*_ is a coherence parameter, *σ*_*c*_ being the source coherence length. The CSD of any SSGS beam at the source can be obtained from Eq. (4) as


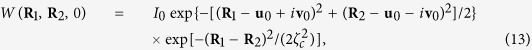


where *I*_0_ is a peak intensity of the beam. The corresponding intensity of SSGS beam can be obtained from Eq. (9) in the form


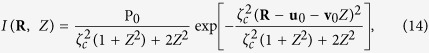


which conforms to the general rule of Eq. (11) with the propagation factor 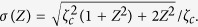 Eq. (14) clearly shows that the linear phase shift ***v***_0_ induces self-steering properties during propagation.

**Figure 1:**
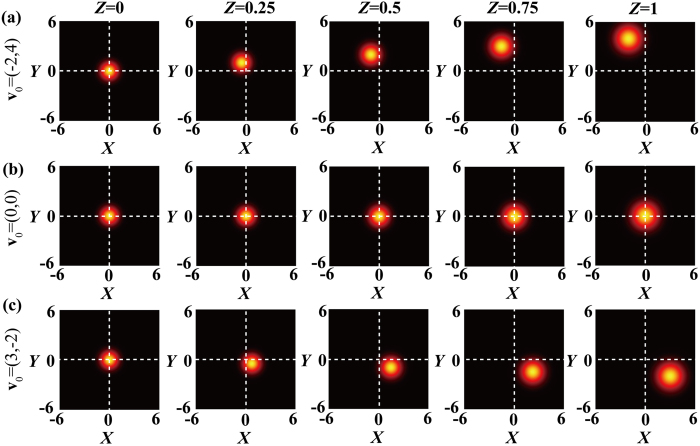
Self-steering of novel beams in free-space. (**a**) SSGS beam intensity distribution at several propagation distances for **v**_0_ = (−2, 4). (**b**) SSGS beam intensity distribution at several propagation distances for **v**_0_ = (0, 0). (**c**) SSGS beam intensity distribution at several propagation distances for ***v***_0_ = (3, −2).

In Fig. 1 we display the SSGS guiding center evolution. In our numerical simulations, we chose the following parameters: *σ*_*I*_ = 1 *mm, σ*_*c*_ = 10 *mm, λ* = 632.8 *nm* and **u**_0_ = (0, 0). It can be inferred from Fig. 1 that the SSGS guiding center can be shifted to any point in the transverse plane of the beam by adjusting ***v***_0_. Thus an SSGS beam can serve as an effective tool for transferring a trapped particle to any desired location as well as for tracking a moving target.

### Experimental generation of self-steering partially coherent beams

**Figure 2:**
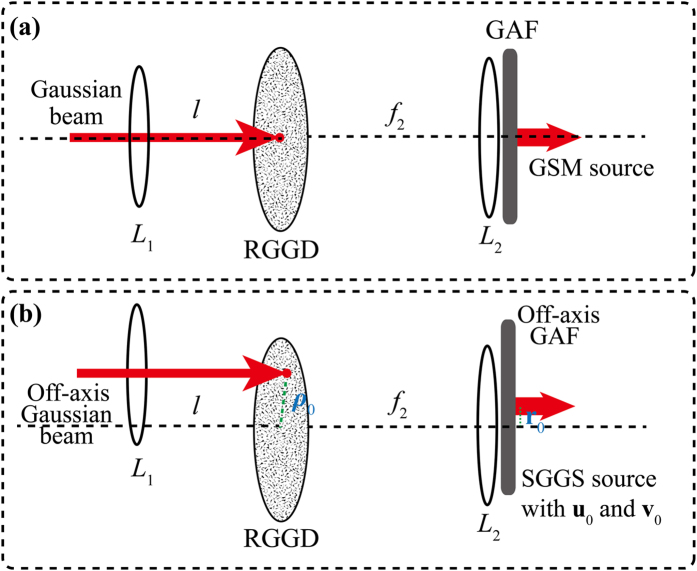
Schematics for generating (**a**) conventional Gaussian Schell-model source, and (**b**) self-steering Gaussian Schell source with controllable parameters **u**_0_ and **v**_0_; *L*_1_ and *L*_2_ thin lenses, rotating ground-glass disk (RGGD), Gaussian amplitude filter (GAF).

Next we show how to generate an SSGS source in practice. As follows from Eq. (13), the CSD of the SSGS source can synthesized by introducing a displacement **u**_0_ and a linear phase shift ***v***_0_ to a conventional GSM source. Figure 2(a) shows a conventional optical system for generating the well-known GSM source, first reported in ref. 20. Figure 2(b) shows our optical system for generating an SSGS source with controllable parameters **u**_0_ and **v**_0_; the latter can be regarded as a modification of the former. In Fig. 2(b), a focused off-axis Gaussian beam with the electric field 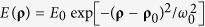 illuminates a rotating ground-glass disk (RGGD), followed by the output beam passing through a thin lens with the focal length *f*_2_ and an off-axis Gaussian amplitude filter (GAF) with the transmission function 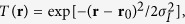 producing an SSGS source. Here **ρ**_0_ = (*ρ*_0*x*_, *ρ*_*oy*_) and *ω*_0_ are the off-axis displacement and the beam waist of the incident Gaussian beam, respectively, **r**_0_ = (*x*_0_, *y*_0_) is the off-axis displacement of the GAF transmission function. In accord with the van Cittert-Zernike theorem^15^, the CSD of the SSGS source in related to the intensity of the incoherent off-axis Gaussian beam (i.e., 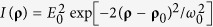) just behind the RGGD as


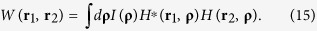


Here *H*(***r***,***ρ***) is the response function of the optical system between the RGGD and the GAF given by^15^,^20^,^21^,^22^


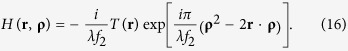


Substituting *I*(**ρ**) and Eq. (16) into Eq. (15), we find that the CSD of the SSGS source has the form of Eq. (13) with the parameters **u**_0_ = **r**_0_/*σ*_*I*_ and **v**_0_ = 2*πσ*_*I*_**ρ**_0_/(*λf*_2_). It follows that the displacement **u**_0_ of the generated SSGS source is related to the off-axis GAF displacement **r**_0_ and the linear phase shift **v**_0_ is determined by the off-axis displacement **ρ**_0_ of the incident off-axis Gaussian beam.

We carry out an experiment to generate and characterize the SSGS beam. In our experiment, the wavelength *λ* = 632.8 *nm*, the focal lengths of thin lenses *L*_1_ and *L*_2_ are equal to 100 *mm* and 200 *mm*, respectively, and the transverse beam width *σ*_*I*_ is equal to 1 *mm*. The off-axis displacement **r**_0_ of the GAF is equal to (0, 0), therefore we generate an SSGS source with **u**_0_ = (0, 0). The generated beam from the SSGS source first passes through a thin lens with the focal length *f* = 400 *mm*, and then arrives at a charge-coupled device (CCD), which measures the focused intensity. The distance between SSGS source and the CCD is equal to *z*. In our experiment, we generate several SSGS beams with different initial coherence widths to examine the coherence impact on the self-steering effect. The initial coherence width is modulated by the beam spot size at the RGGD. In particular, we generate three SSGS sources with the initial coherence widths *σ*_*c*_ = 1.0 *mm*, 0.5 *mm* and 0.2 *mm*, respectively. The experimental techniques for measuring the degree of coherence can be found in refs 23, 24, 25.

**Figure 3:**
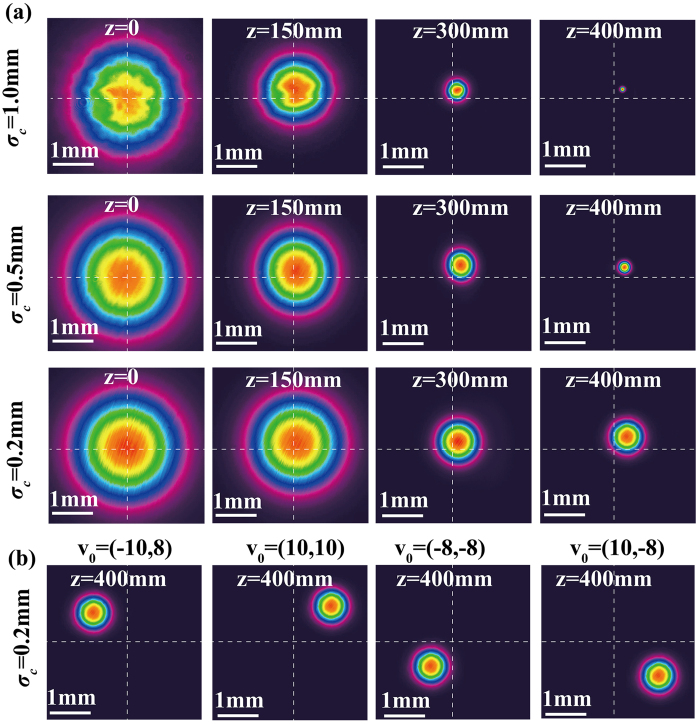
Experimental evidence of the beam guiding center evolution. (**a**) SSGS beam intensity distribution at several propagation distances given the same **v**_0_ = (5, 5) and different values of the initial coherence width. (**b**) Same as in (**a**) in the focal plane with *σ*_*c*_ = 0.2 *mm* and different values of **v**_0_.

**Figure 4:**
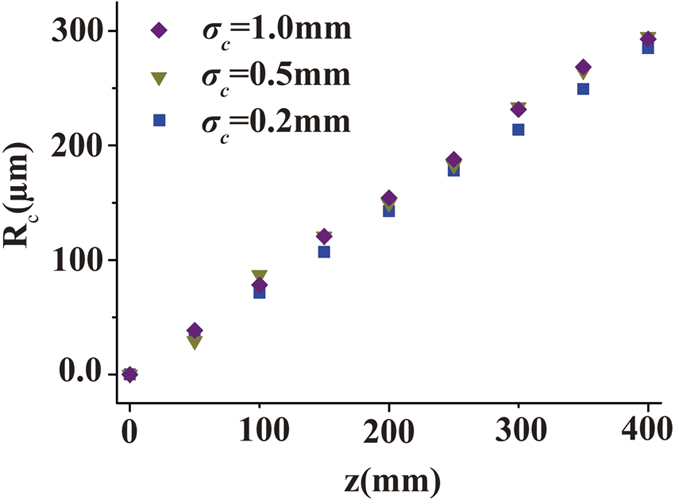
Guiding center position **R**_*c*_ as a function of the propagation distance *z* for three cases (*σ*_*c*_ = 1.0 *mm, σ*_*c*_ = 0.5 *mm, σ*_*c*_ = 0.2 *mm*) with the same **v**_0_ = (5, 5).

We display the experimental results in Fig. 3. Figure 3(a) shows the SSGS beam intensity distribution with the same **v**_0_ = (5, 5) for different *σ*_*c*_ at several propagation distances, while Fig. 3(b) shows the same quantity for *σ*_*c*_ = 0.2 *mm* in the focal plane for different values of **v**_0_. It can be inferred from Fig. 3 that the guiding center motion can be controlled by adjusting the linear phase shift ***v***_0_ at the source. We stress that although the apparent beam focusing in the figure is caused by the lens, the lens is not essential for beam self-steering. The latter results from the linear phase shift imparted to the beam at the source; the phase shift can be imprinted without a lens by using, for instance, a phase mask. The lens in our experimental setup serves primarily for convenience of imaging. To quantitatively ascertain the beam coherence width effect on the guiding center dynamics, visualized in Fig. 3(a), we present in Fig. 4 the SSGS beam guiding center position as a function of the propagation distance *z* for three values of coherence width (*σ*_*c*_ = 1.0 *mm*,* σ*_*c*_ = 0.5 *mm, σ*_*c*_ = 0.2 *mm*). We find that the initial coherence state of the beam has virtually no effect on its guiding center dynamics making our experimental results consistent with the theory, *c.f.*, Eq. (14).

**Figure 5:**
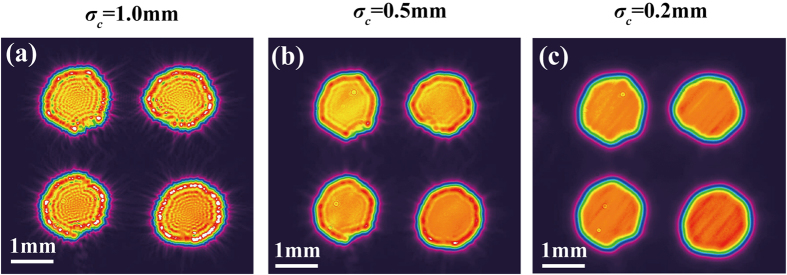
SSGS beam intensity distribution as the beam passes through a 2 × 2 circle hole array in three cases corresponding to different initial coherence widths.

Figure 5 shows a measured SSGS beam intensity distribution as the beam passes through a 2 × 2 circular hole array for three cases corresponding to different source coherence states. It is found from Fig. 5 that the higher the beam coherence the greater the intensity profile distortion due to speckles and spurious fringes, making SSGS beams of low coherence preferable for trapped particle transport/manipulations and mobile target tracing.

## Discussion

Finally, we show that the straight line guiding center trajectory is a generic property of any self-steering beams, either shape-invariant or not, rooted in the linear momentum conservation in free space. The simplest, and, perhaps, most elegant proof relies on the established analogy between paraxial beam propagation and Hilbert space time evolution of a quantum particle with the beam propagation distance being analogous to time; see, for instance refs 26 and 27. The coordinate and linear momentum of the equivalent quantum particle are analogous to the beam guiding center position angular spread, respectively. One can then show, following these references, that free space paraxial beam propagation is equivalent to free quantum particle evolution which conserves linear momentum. As a result, the beam guiding center position must be either static or evolve linearly with the propagation distance, implying a straight line trajectory.

In summary, we have introduced a class of partially coherent beams with their guiding centers propagating along straight line trajectories, controlled by the linear phase shift at the source. We generate the new beams experimentally and confirm all our theoretical predictions. The new beams are anticipated to find applications for trapped particle transport and mobile target tracing.

## Additional Information

**How to cite this article**: Chen, Y. *et al*. Self-steering partially coherent beams. *Sci. Rep.* **7**, 39957; doi: 10.1038/srep39957 (2017).

**Publisher's note:** Springer Nature remains neutral with regard to jurisdictional claims in published maps and institutional affiliations.

## References

[b1] Ashkin A. Acceleration and Trapping of Particles by Radiation Pressure. Phys. Rev. Lett. 24, 156–159 (1970).

[b2] Ashkin A., Dziedzic J. M., Bjorkholm J. E. & Chu S. Observation of a single-beam gradient force optical trap for dielectric particles. Opt. Lett. 11, 288–290 (1986). 19730608 10.1364/ol.11.000288

[b3] Grier D. G. A revolution in optical manipulation. Nature 424, 810–816 (2003). 12917694 10.1038/nature01935

[b4] Neuman K. C. & Block S. M. Optical trapping. Rev. Sci. Instrum. 75, 2787–2809 (2004). 16878180 10.1063/1.1785844 PMC1523313

[b5] Oroszi L., Galajda P., Kirei H., Bottka S. & Ormos P. Direct measurement of torque in an optical trap and its application to double-strand DNA. Phys. Rev. Lett. 97, 058301 (2006). 17026144 10.1103/PhysRevLett.97.058301

[b6] Maragò O. M., Jones P. H., Gucciardi P. G., Volpe G. & Ferrari A. C. Optical trapping and manipulation of nanostructures. Nat. Nanotechnol. 8, 807–819 (2013). 24202536 10.1038/nnano.2013.208

[b7] Collett E. & Wolf E. Is complete spatial coherence necessary for the generation of highly directional light beams? Opt. Lett. 2, 27–29 (1978). 19680395 10.1364/ol.2.000027

[b8] Wolf E. & Collett E. Partially coherent sources which produce the same far-field intensity distribution as a laser. Opt. Commun. 25, 293–296 (1978).

[b9] Auñón J. M. & Nieto-Vesperinas M. Partially coherent fluctuating sources that produce the same optical force as a laser beam. Opt. Lett. 38, 2869–2872 (2013). 23903166 10.1364/OL.38.002869

[b10] Ponomarenko S. A., Huang W. & Cada M. Dark and antidark diffraction-free beams. Opt. Lett. 32, 2508–2510 (2007). 17767287 10.1364/ol.32.002508

[b11] Zhao C., Cai Y., Lu X. & Eyyuboglu H. T. Radiation force of coherent and partially coherent flat-topped beams on a Rayleigh particle. Opt. Express 17, 1753–1765 (2009). 19189005 10.1364/oe.17.001753

[b12] Zhao C. & Cai Y. Trapping two types of particles using a focused partially coherent elegant Laguerre–Gaussian beam. Opt. Lett. 36, 2251–2253 (2011). 21685983 10.1364/OL.36.002251

[b13] Chen Y. & Cai Y. Generation of a controllable optical cage by focusing a Laguerre-Gaussian correlated Schell model beam. Opt. Lett. 39, 2549–2552 (2014). 24784042 10.1364/OL.39.002549

[b14] Liu X. & Zhao D. Trapping two types of particles with a focused generalized Multi-Gaussian Schell model beam. Opt. Commun. 354, 250–255 (2015).

[b15] Mandel L. & Wolf E. Optical Coherence and Quantum Optics (Cambridge University Press, 1995).

[b16] Barenblatt G. I. Scaling, self-similarity, and intermediate asymptotics (Cambridge University Press, 1996).

[b17] Ponomarenko S. A. Complex Gaussian representation of statistical pulses. Opt. Express 19, 17086–17091 (2011). 21935069 10.1364/OE.19.017086

[b18] Ma L. & Ponomarenko S. A. Optical coherence gratings and lattices. Opt. Lett. 39, 6656–6659 (2014). 25490645 10.1364/OL.39.006656

[b19] Ma L. & Ponomarenko S. A. Free-space propagation of optical coherence lattices and periodicity reciprocity. Opt. Express 23, 1848–1856 (2015). 25835938 10.1364/OE.23.001848

[b20] De Santis P., Gori F., Guattari G. & Palma C. An example of a Collett-Wolf source. Opt. Commun. 29, 256–260 (1979).

[b21] Goodman J. Introduction to Fourier Optics (Roberts and Company, 2005).

[b22] Wang F., Liu X., Yuan Y. & Cai Y. Experimental generation of partially coherent beams with different complex degrees of coherence. Opt. Lett. 38, 1814–1816 (2013). 23722753 10.1364/OL.38.001814

[b23] Chen Y., . Generation and propagation of a partially coherent vector beam with special correlation functions. Phys. Rev. A 89, 013801 (2014).

[b24] Cai Y., Chen Y. & Wang F. Generation and propagation of partially coherent beams with nonconventional correlation functions: a review [invited]. J. Opt. Soc. Am. A 31, 2083–2096 (2014). 10.1364/JOSAA.31.002083 25401450

[b25] Chen Y., Gu J., Wang F. & Cai Y. Self-splitting properties of a Hermite-Gaussian correlated Schell-model beam. Phys. Rev. A 91, 013823 (2015).

[b26] Ponomarenko S. A., Greffet J. J. & Wolf E. The diffusion of partially coherent beams in turbulent media. Opt. Commun. 128, 1–8 (2002).

[b27] Ponomarenko S. A. Self-imaging of partially coherent light in graded-index media. Opt. Lett. 40, 566–568 (2015). 25680151 10.1364/OL.40.000566

